# NOD2/c-Jun NH_2_-Terminal Kinase Triggers Mycoplasma ovipneumoniae-Induced Macrophage Autophagy

**DOI:** 10.1128/JB.00689-19

**Published:** 2020-09-23

**Authors:** Haixia Luo, Xixi Wu, Zhaokun Xu, Xiujing Hao, Yongyu Wang, Min Li

**Affiliations:** a Life Science School, Ningxia University, Yinchuan, Ningxia Hui Autonomous Region, China; b Key Laboratory of Ministry of Education for Conservation and Utilization of Special Biological Resources in the Western China, Ningxia University, Yinchuan, Ningxia Hui Autonomous Region, China; Brigham and Women's Hospital/Harvard Medical School

**Keywords:** *Mycoplasma ovipneumoniae*, NOD2, autophagy, JNK pathway

## Abstract

Mycoplasma ovipneumoniae belongs to *Mycoplasma*, a genus containing the smallest self-replicating microorganisms, and causes infectious pleuropneumonia in goats and sheep. Nucleotide-binding oligomerization domain-containing protein (NOD2), an intracellular pattern recognition receptor, interacts with muramyl dipeptide (MDP) to recognize bacterial peptidoglycans and is involved in autophagy induction. However, there have been no reports about NOD recognition of mycoplasmas or M. ovipneumoniae-induced autophagy. In this study, we sought to determine the role of NOD2 in M. ovipneumoniae-induced autophagy using Western blotting, immunofluorescence, real-time PCR (RT-PCR), and color-changing unit (CCU) analysis. M. ovipneumoniae infection markedly increased NOD2 but did not increase NOD1 expression in RAW 264.7 cells. Treating RAW 264.7 cells with MDP significantly increased colocalization of M. ovipneumoniae and LC3, whereas treatment with NOD inhibitor, NOD-IN-1, decreased colocalization of M. ovipneumoniae and LC3. Furthermore, suppressing NOD2 expression with small interfering RNA (siRNA)-NOD2 failed to trigger M. ovipneumoniae-induced autophagy by detecting autophagy markers Atg5, beclin1, and LC3-II. In addition, M. ovipneumoniae infection significantly increased the phosphorylated c-Jun NH_2_-terminal kinase (p-JNK)/JNK, p-Bcl-2/Bcl-2, beclin1, Atg5, and LC3-II ratios in RAW 264.7 cells. Treatment with JNK inhibitor, SP600126, or siRNA-NOD2 did not increase this reaction. These findings suggested that M. ovipneumoniae infection activated NOD2, and both NOD2 and JNK pathway activation promoted M. ovipneumoniae-induced autophagy. This study provides new insight into the NOD2 reorganization mechanism and the pathogenesis of M. ovipneumoniae infection.

**IMPORTANCE** M. ovipneumoniae, which lacks a cell wall, causes infectious pleuropneumonia in goats and sheep. In the present study, we focused on the interaction between NOD and M. ovipneumoniae, as well as its association with autophagy. We showed for the first time that NOD2 was activated by M. ovipneumoniae even when peptidoglycans were not present. We also observed that both NOD2 and JNK pathway activation promoted M. ovipneumoniae-induced autophagy.

## Abstract (precis)

M. ovipneumoniae, which lacks a cell wall, causes infectious pleuropneumonia in goats and sheep. In the present study, we focused on the interaction between NOD and M. ovipneumoniae, as well as its association with autophagy. We showed for the first time that NOD2 was activated by M. ovipneumoniae even when peptidoglycans were not present. We also observed that both NOD2 and JNK pathway activation promoted M. ovipneumoniae-induced autophagy.

## INTRODUCTION

Mycoplasma species are wall-less microorganisms with minute genomes that are believed to have evolved by degenerative evolution from *Firmicutes* (1, 2). Mycoplasmas are widespread in the natural world as important parasites of humans, mammals, reptiles, fish, arthropods, and plants (3). Among these species, Mycoplasma ovipneumoniae is the causative agent of chronic, nonprogressive pneumonia, which causes infectious pleuropneumonia in goats and sheep (4). M. ovipneumoniae has become a worldwide livestock disease and a direct threat to the sheep industry; thus far, no efficient or safe vaccine has been found (4–6).

Nucleotide-binding oligomerization domain (NOD)-containing proteins NOD1 and NOD2 are two well-characterized intracytosolic pattern recognition receptors (PRRs), which are members of the intracellular NOD-like receptor (NLR) family. NOD1 and NOD2 are homologous proteins. NOD1 contains a single N-terminal caspase recruitment domain (CARD), while two CARD domains are found in NOD2. Centrally located nucleotide-binding domains (NBDs) and C-terminal leucine-rich repeat domains (LRRs) are found in both NOD1 and NOD2 (7, 8). The CARD interacts with downstream adaptor proteins, which are required for proinflammatory signaling pathways (9). The NBD mediates interactions required for homo-oligomer formation, and the LRR is involved in peptidoglycan recognition (10, 11). Muramyl dipeptide (MDP), the smallest structural subunit of bacterial peptidoglycan, has been proven to be the direct contact between MDP and NOD2 (12). NOD proteins recognize MDP and initiate host immune response (13, 14). Upon recognition of MDP, NOD2 undergoes self-oligomerization and activates serine-threonine kinase receptor-interacting protein 2 (RIP2) (12). However, there have been no studies about NOD recognition of mycoplasma when peptidoglycan is not present.

Autophagy is a highly conserved process of cellular self-digestion in which excessive, damaged, or aged proteins and intracellular organelles are sequestered in double-membraned vesicles of autophagosomes and then terminally self-digested in lysosomes (15, 16). Numerous other conditions can trigger autophagy, including hypoxia, oxidative stress, and radiation (17). In recent years, many studies have demonstrated that intracellular bacteria can also be targeted by autophagic degradation in a process termed xerophagy. The bacteria in the cytoplasm can be recognized and captured by autophagic machinery and can thereby eventually be targeted by lysosomes (18). The c-Jun NH_2_-terminal kinase (JNK) signal transduction pathway is linked to the molecular events involved in autophagy regulation (19). JNK activation can phosphorylate Bcl-2 and then, in turn, degrade Bcl-2 and dissociate beclin1 from the beclin1/Bcl-2 complex, leading to induction of autophagy (20, 21). It has been demonstrated that cytoadherence of M. pneumoniae in the respiratory tract is the initial event in infection. This event is mediated by P1 adhesin and other proteins, which induce inflammatory responses through Toll-like receptor 4 (TLR4) and autophagy (22). However, the role of JNK signaling in M. ovipneumoniae-induced autophagy has not been elucidated.

In the present study, we investigated the role of NOD2 in regulating M. ovipneumoniae infection. We provide evidence that NOD2 can be activated by M. ovipneumoniae infection and that NOD2 activation enhances M. ovipneumoniae-induced autophagy. These effects were mediated by the JNK signaling pathway.

## RESULTS

### NOD2 is involved in M. ovipneumoniae-induced macrophage autophagy.

**FIG 1:**
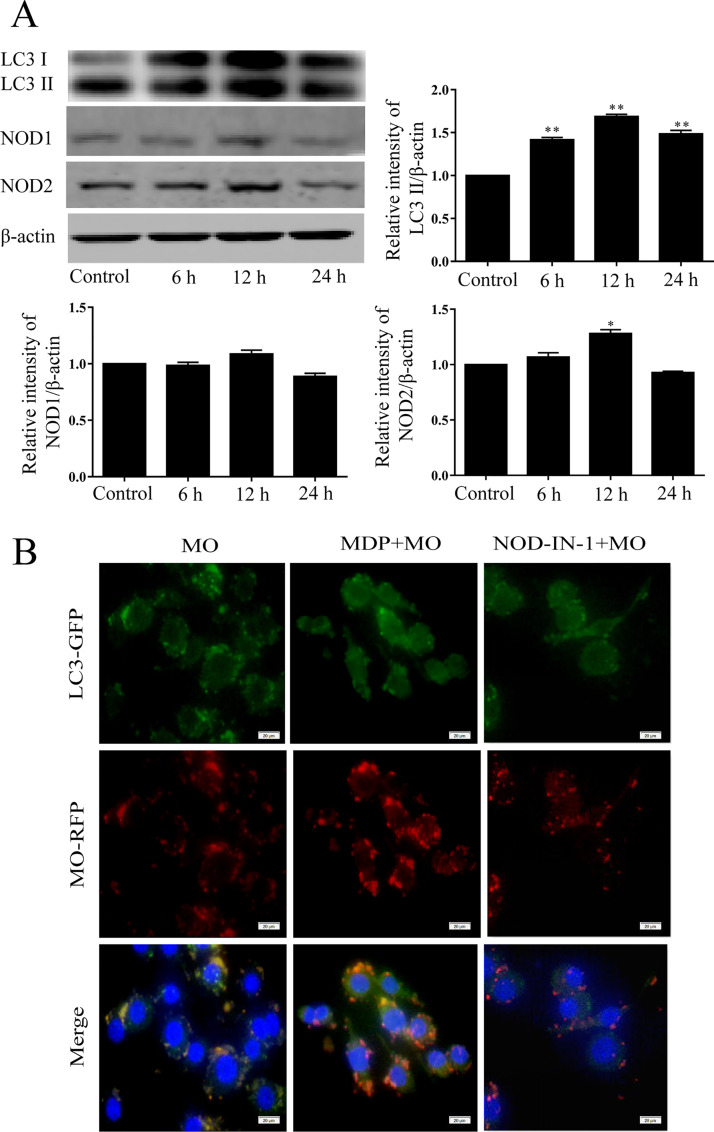
NOD2-involved M. ovipneumoniae-induced macrophage autophagy. (A) Expression of NOD2 and LC3 were measured by Western blotting in RAW 264.7 cells postinfection with M. ovipneumoniae (MOI of 10) for 6 h, 12 h, and 24 h. Values are shown as the mean ± SD; *n* = 3 (*, *P* ≤ 0.05; **, *P* ≤ 0.01). (B) Colocation of M. ovipneumoniae and LC3 in RAW 264.7 cells, which were treated with MDP (50 μg/ml) or NOD-IN-1 (25 μM) and then infected with M. ovipneumoniae (MOI of 10) for 12 h. M. ovipneumoniae was stained with Dil in red; LC3 was stained with anti-LC3 antibody and FITC-labeled secondary antibody (green). DNA of macrophage was stained with DAPI (blue). Scale bars, 20 μm. Values are shown as the mean ± SD; *n* = 3.

Transformation of LC3-I to LC3-II is commonly used as a specific marker for autophagosome formation (23). NOD1 and NOD2 are viewed as cytosolic sensors of bacterial peptidoglycan fragments (10, 24). To test the involvement of NOD1/NOD2 in M. ovipneumoniae-induced macrophage autophagy, we measured NOD1, NOD2, and LC3-II levels in RAW 264.7 cells following M. ovipneumoniae infection in a time-dependent manner. Western blot analysis demonstrated that in Raw264.8 cells, LC3-II was significantly increased at 6 h, 12 h, and 24 h after M. ovipneumoniae infection, and NOD2 was significantly increased at 12 h, results that were not seen in uninfected cells (control). However, there was no difference in NOD1 expression in M. ovipneumoniae-infected RAW 264.7 cells compared with the control (Fig. 1A). These results suggest that NOD2 may be involved in M. ovipneumoniae-induced RAW 264.7 autophagy, while NOD1 is not.

MDP is an activator of NOD2, and NOD-IN-1 is a potent mixed inhibitor of NOD1 and NOD2 (25). To further test the involvement of NOD2 in M. ovipneumoniae-induced autophagy, we measured the colocalization of M. ovipneumoniae and LC3 in RAW 264.7 cells treated with MDP or NOD-IN-1. RAW 264.7 cells were treated with MDP (50 μg/ml) or NOD-IN-1 (25 μM) and then infected with M. ovipneumoniae for 12 h, after which the localization of M. ovipneumoniae and autophagy marker protein LC3 was observed using confocal microscopy. M. ovipneumoniae was stained with Dil in red, while LC3, which represents the autophagosome, is labeled in green. M. ovipneumoniae was observed as small red particles in the macrophage and was colocalized with LC3 in infected RAW 264.7 cells. MDP pretreatment further increased the colocalization of M. ovipneumoniae and LC3. Additionally, the NOD-IN-1 inhibitor significantly attenuated the colocalization between M. ovipneumoniae and LC3 (Fig. 1B).

**FIG 2:**
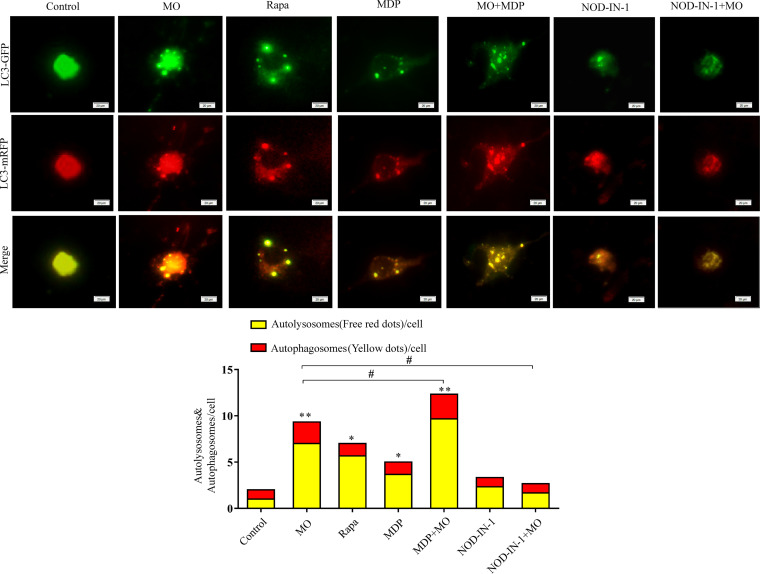
NOD2 activation enhanced M. ovipneumoniae-induced autophagic flux. RAW 264.7 cells were transfected with mRFP-GFP-LC3A/B (red and green) followed by M. ovipneumoniae infection (MOI of 10) and/or rapamycin (50 μg/ml), MDP (50 μg/ml), or NOD-IN-1 (25 μM) treatment for 12 h. The amount of autophagosome (yellow) and autolysosome (red) per cell was determined using Impairs software. Scale bars, 20 μm. *, *P* < 0.05; **, *P* < 0.05 versus control group; *#*, *P* < 0.05 versus M. ovipneumoniae group. The data represent the mean ± SD from three independent experiments performed in triplicate.

Adenovirus mRFP-GFP-LC3 (monomeric red fluorescent protein-green fluorescent protein-LC3) was used to monitor and measure the autophagic flux rate, while RFP-positive (RFP^+^) GFP^+^ puncta detected autophagosomes, and RFP^+^ GFP^−^ puncta detected quenched signaling of RFP in acidic autolysosomes (25). In the present study, mRFP-GFP-LC3 was used to evaluate the effect of NOD2 on M. ovipneumoniae-induced autophagic flux. According to mRFP-GFP-LC3 puncta formation assays, both yellow puncta (autophagosomes) and red puncta (autolysosomes) were higher in M. ovipneumoniae-infected cells, cells receiving rapamycin (Rap) treatment, cells receiving MDP treatment, and the MDP plus M. ovipneumoniae group than the control (*P < *0.05). No significant difference was found among cells receiving NOD-IN-1 treatment, NOD-IN-1 plus M. ovipneumoniae treatment, and the control group (Fig. 2). This observation suggested that NOD2 activation enhanced M. ovipneumoniae-induced autophagic flux.

### Suppressing NOD2 with NOD2 siRNA decreases autophagy induced by M. ovipneumoniae.

**FIG 3:**
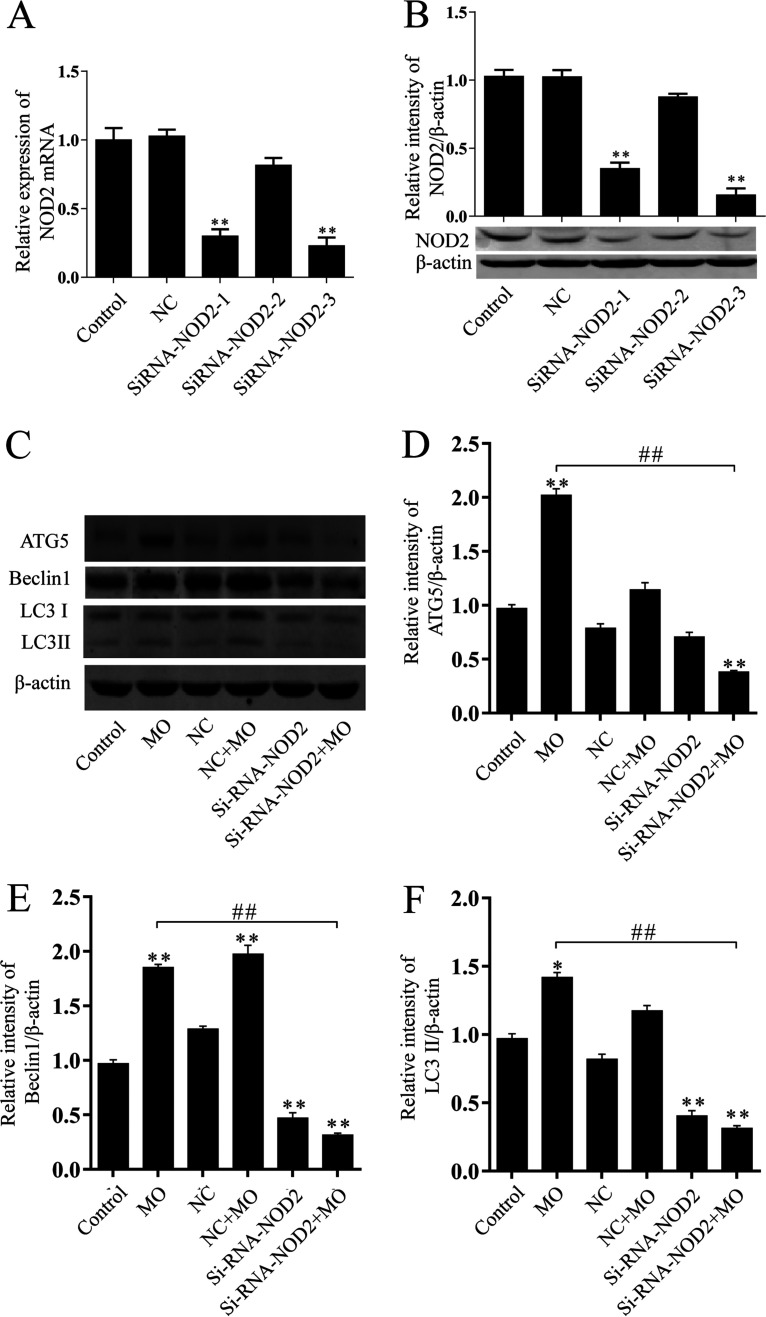
Suppressing NOD2 decreased autophagy induced by M. ovipneumoniae. (A and B) RAW 264.7 cells were transfected with pMCSV-Si-RNA-NOD2-1/2/3 for 24 h. NOD2 mRNA level and protein expression were analyzed by RT-PCR (A) and Western blotting (B). (C to F) RAW 264.7 cells were transfected with pMCSV-si-RNA-NOD2-3 or an equal amount of empty vector followed by M. ovipneumoniae infection (MOI of 10) for 12 h. The expression of Atg5, beclin1, and LC3 was analyzed by Western blot analysis. Values are shown as the mean ± SD; *n* = 3 (* and #, *P* ≤ 0.05; ** and ##, *P* ≤ 0 .01).

NOD-IN-1 is a mixed inhibitor of NOD1 and NOD2 (25). To further verify the impact of NOD2 on M. ovipneumoniae-induced macrophage autophagy, RAW 264.7 cells were transfected with small interfering (siRNA)-control (NC) or siRNA-NOD2 and then infected with M. ovipneumoniae for 12 h. A decrease in NOD2 was observed after transfection with siRNA-NOD2-1 and siRNA-NOD3-3. siRNA-NOD2-3 had the highest inhibitory efficiency for DOD2 among the three siRANA-NOD2 groups (Fig. 3A and B). Therefore, Si-RNA-NOD2-3 was used to inhibit the expression of NOD2, written as siRNA-NOD2.

LC3, Atg5, and beclin1 are autophagy marker proteins that have been widely used to evaluate the level of autophagy. LC3 is a ubiquitin-like protein that plays an important role in autophagosome membrane formation and expansion (23). Atg5 is a key autophagy protein required for the conjugation of LC3 (23), while beclin1 plays an important role in the autophagic process (26). LC3-II, beclin1, and Atg5 were examined by Western blotting. M. ovipneumoniae-infected RAW 264.7 cells had significantly increased levels of LC3-II, beclin1, and Atg5 compared with the control or NC groups. Downregulation of NOD2 by siRNA markedly suppressed the M. ovipneumoniae-induced activation of LC3-II, beclin1, and Atg5 (Fig. 3C). Thus, taking these results together, we concluded that M. ovipneumoniae infection can activate NOD2 and that NOD2 activation, in turn, promoted M. ovipneumoniae-induced autophagy.

### M. ovipneumoniae promotes autophagy via the JNK signaling pathway.

**FIG 4:**
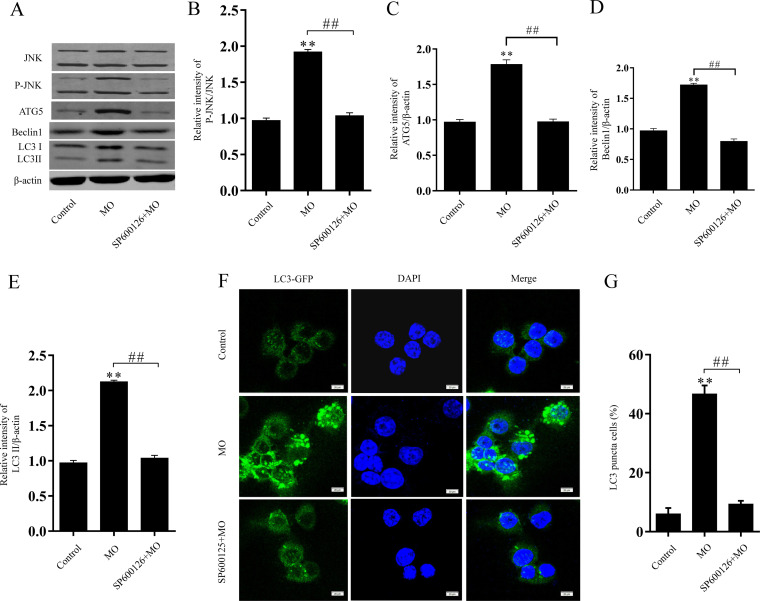
JNK activation promoted M. ovipneumoniae-induced autophagy. (A to E) Western blot analysis determined protein levels of JNK, p-JNK, Atg5, beclin1, and LC3 in RAW 264.7 cells with M. ovipneumoniae infection (MOI of 10) and/or SP600125 treatment (20 μM). (F and G) Autophagosome accumulation revealed by LC3-II accumulation in RAW 264.7 cells with M. ovipneumoniae infection (MOI of 10) and/or SP600125 treatment. Scale bars, 20 μm. Values are shown as the mean ± SD; *n* = 3 (** and ##, *P* ≤ 0.01).

**FIG 5:**
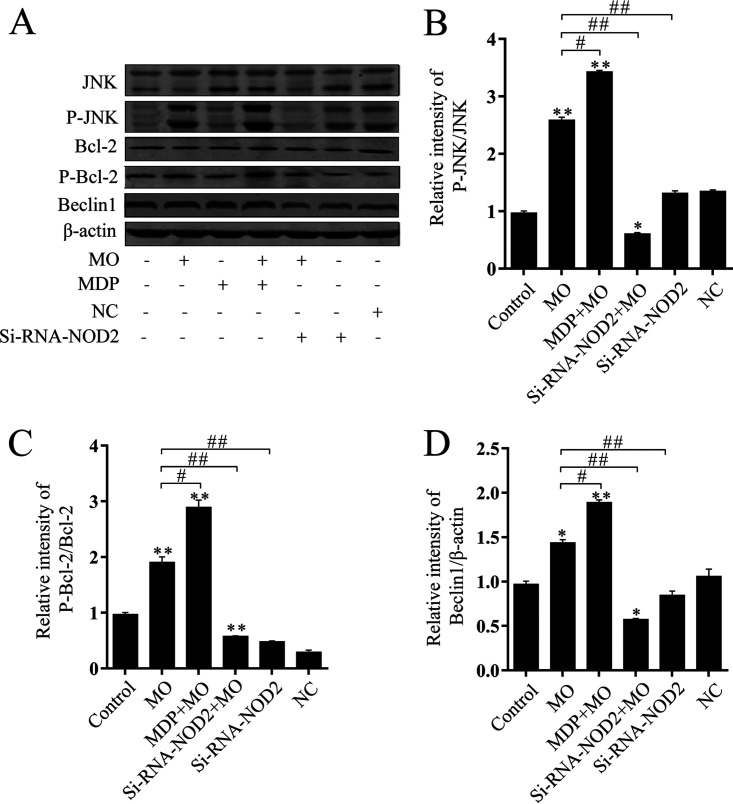
NOD2 deficiency reduced JNK activation in M. ovipneumoniae-infected RAW 264.7 cells. Shown are representative Western blotting and summarized data showing the protein levels of JNK, p-JNK, Bcl-2, p-Bcl-2, and beclin1 expression in RAW 264.7 cells transfected with siRNA-NOD2 or stimulated with MDP (50 μg/ml) followed by M. ovipneumoniae infection (MOI of 10) for 12 h. Values are shown as the mean ± SD; *n* = 3 (* and *#*, *P* ≤ 0.05; ** and ##, *P* ≤ 0.01).

The c-Jun NH_2_-terminal kinase (JNK) pathway plays an important role in various forms of autophagy (27). JNK-dependent phosphorylation of Bcl-2 antiregulation beclin1 protein level dissociates the beclin1/Bcl-2 complex and degrades Bcl-2, leading to both apoptosis and autophagy (19, 28). SP600126 is a JNK inhibitor (29). Western blot analysis was used to investigate whether M. ovipneumoniae infection had an effect on JNK stimulation in RAW 264.7 cells. The phosphorylation levels of p-JNK, p-Bcl-2, and beclin1 were significantly increased in RAW 264.7 cells following M. ovipneumoniae infection compared with control cells (Fig. 4A and 5). Inhibiting JNK with SP600126 in RAW 264.7 cells markedly decreased the activation of LC3II, beclin1, and Atg5 in RAW 264.7 cells 12 h after M. ovipneumoniae infection (Fig. 4A). Similar results were observed by testing GFP-LC3 in RAW 264.7 cells following M. ovipneumoniae infection and sp600126 treatment. A large amount of punctate GFP-LC3 was observed in infected RAW 264.7 cells. In contrast, treatment with sp600126 prior to the M. ovipneumoniae infection resulted in the failure of a robust increase of GFP-LC3 in RAW 264.7 cells (Fig. 4F). These findings suggested that M. ovipneumoniae induced macrophage autophagy by activating the JNK pathway.

### NOD2/JNK is required for M. ovipneumoniae-induced macrophage autophagy.

Since the above results demonstrated that M. ovipneumoniae infection could activate the JNK pathway, we further examined whether NOD2 associated with this pathway. We observed that the downregulation of NOD2 by siRNA markedly suppressed the M. ovipneumoniae-induced activation of p-JNK, p-Bcl-2, and beclin1 in RAW 264.7 cells (Fig. 5). These findings suggested that both NOD2 and JNK pathway activation were required for autophagy in M. ovipneumoniae-infected cells.

### NOD2 activation defends against M. ovipneumoniae proliferation in RAW 264.7 cells.

**FIG 6:**
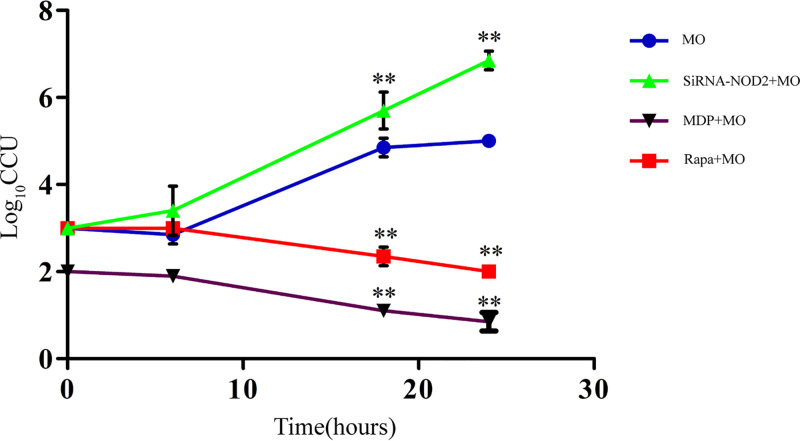
Amount of intracellular M. ovipneumoniae. After the addition of SiRNA-NOD2, MDP (50 μg/ml), or rapamycin (50 μg/ml), the intracellular M. ovipneumoniae (MOI of 10) burden was measured at 6 h, 18 h, and 24 h postinfection. RAW 264.7 cells were lysed, and intracellular bacteria were evaluated through CCU by performing serial dilution of macrophage lysates. Values are shown as the mean ± SD; *n* = 3 (*, *P* ≤ 0.05; **, *P* ≤ 0.01). MO, M. ovipneumoniae.

To explore the effect of NOD2 activation on M. ovipneumoniae intracellular survival, siRNA, MDP, and rapamycin were used in this study. We detected the intracellular proliferation of M. ovipneumoniae at 6 h, 18 h, and 24 h postinfection. As presented in Fig. 6, compared to the M. ovipneumoniae infection group, the M. ovipneumoniae load in the siRNA group was considerably increased at 18 h and 24 h postinfection. Moreover, intracellular M. ovipneumoniae was significantly decreased in the MDP and rapamycin treatment groups. This proved that NOD2 activation limited M. ovipneumoniae proliferation in macrophages.

## DISCUSSION

It is well-known that peptidoglycan is detected intracellularly by NOD1 and NOD2. NOD1 recognizes γ-d-glutamyl-mesodiaminopimelic acid in Gram-negative and Gram-positive bacteria (24, 30), while NOD2 detects MDP structures in both Gram-negative and Gram-positive bacteria (13, 31). Increasing evidence suggests that NOD, in addition to recognizing peptidoglycan, can also sense peptidoglycan-independent proteins or pathogens (32–34). For instance, the effector proteins IPGb2 and OspB of Shigella flexneri and SipA and SopE of Salmonella enterica serovar Typhimurium were found to activate the NOD1/NOD12 signaling pathway in a peptidoglycan-independent manner (32, 34). In addition, viruses and parasites have also provided evidence for additional peptidoglycan-independent roles of NOD1 and NOD2 (10). During respiratory syncytial virus infection, NOD2, but not NOD1, can sense single-stranded RNA (ssRNA) from the virus and interacts with mitochondrial antiviral signaling (MAVS) (35, 36). Parasites are pathogens that lack peptidoglycan. In Plasmodium berghei infection, mice deficient in NOD1 and NOD2 exhibited a decreased inflammatory cytokine response (37). Mycoplasmas are self-replicating organisms without a cell wall; instead, the cells are surrounded by cell membranes. The unique components of peptidoglycan, muramic acids, and diaminopimelic acids could not be detected in mycoplasmas. In this study, we present the first evidence that NOD2 expression is increased during M. ovipneumoniae infection in macrophages but NOD1 expression is not. Activation of NOD2 by MDP enhanced M. ovipneumoniae-induced autophagy. In contrast, downregulation of NOD2 by NOD inhibitor or NOD2-siRNA attenuated these effects (Fig. 1 to 3). We suggest that as in viral infection, M. ovipneumoniae infection can activate NOD2 but not NOD1. Although mycoplasma species do not have a cell wall, an abundance of cell surface antigens and many putative lipoprotein-encoding genes have been found in sequenced mycoplasma genomes (38, 39). For example, mycoplasma cells do not contain TLR ligands, but the purified or synthesized lipoproteins of mycoplasma species induce inflammatory responses through TLR2 (40, 41). Lipoproteins derived from various mycoplasma species have been reported to act as pathogen-associated molecular patterns (PAMPs) (42). Therefore, these cell surface antigens and putative mycoplasma lipoproteins may act as ligands to activate NOD2.

Autophagy is an effective internal regulatory mechanism that allows a biological organism to adapt to different environments. It can protect organisms from metabolic stress, acting as a “housekeeper” (18). In recent years, autophagy has been considered a part of innate immunity against intracellular pathogens (16). The pathogens can be recognized and captured by autophagic machinery. However, several microorganisms have developed a mechanism to escape degradation through autophagy, or even to inhibit autophagy (18, 43). LC3, Atg5, and beclin1, autophagy marker proteins, have been widely used to evaluate the level of autophagy. LC3 is considered to be an important mediator during autophagic vesicle trafficking. Atg5 and Atg7 play significant roles in the Atg12 conjugation system and LC3 (44). c-Jun NH_2_-terminal kinase (JNK) activation has been found to be involved in various forms of autophagy (27). JNK regulates autophagy by phosphorylating Bcl-2 and releasing its binding from beclin1. Beclin1 becomes available for the formation of the Bcl-2/beclin1 complex. This process leads to autophagy (19, 28). However, there have been no studies regarding whether JNK activation is involved in regulating mycoplasma-induced autophagy. The present study showed that M. ovipneumoniae induced JNK activation and autophagy. Inhibition of JNK activation by a specific JNK inhibitor blocked M. ovipneumoniae-induced autophagy. These observations collectively suggest that JNK activation plays a critical role in inducing complete autophagy (Fig. 4 and 5).

**FIG 7:**
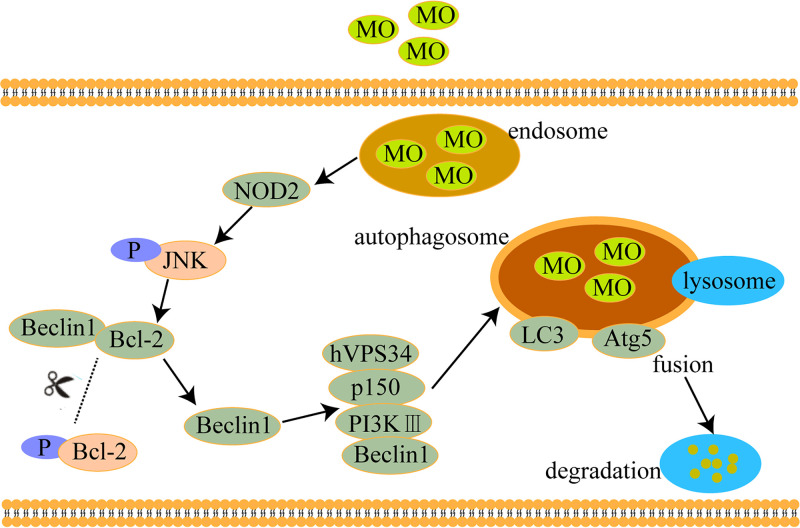
Model of how NOD2/JNK triggers M. ovipneumoniae-induced autophagy.

Autophagy is activated primarily by NOD sensors of innate immunity (45). Several studies have shown that a physical interaction between NOD2 and ATG16L1 is required for the autophagic clearance of intracellular pathogens (46) and that both gene variants are defective in implementing proper autophagy (47). The current study demonstrated that p-JNK, p-BclII, and beclin1 were upregulated in M. ovipneumoniae-infected RAW 264.7 cells. Whereas treatment with JNK inhibitor or Si-NOD2 attenuated these effects, the results indicated that both NOD2 and JNK activation were required for M. ovipneumoniae-induced autophagy and that the absence of either component prevented proper autophagy (Fig. 5). We also analyzed the effect of NOD2 on the intracellular survival of M. ovipneumoniae. We observed that reduced survival of intracellular M. ovipneumoniae was specifically associated with NOD2-induced autophagic-killing (Fig. 6). The graphical diagram of this study is shown in Fig. 7. In summary, we can conclude that M. ovipneumoniae activates NOD2 and that NOD2 and the JNK pathway mediate M. ovipneumoniae-induced autophagy.

## MATERIALS AND METHODS

### Reagents and antibodies.

Dulbecco’s modified Eagle’s medium (DMEM) and fetal bovine serum (FBS) were purchased from HyClone (Logan, UT). Rapamycin, muramyl dipeptide (MDP), NO-IN-1, and sp600126 were purchased from MedChemExpres (NJ). The antibodies anti-β-actin, anti-LC3, anti-NOD1, anti-NOD2, anti-Atg5, anti-beclin1, anti-JNK1/2, anti-p-JNK1/2, anti-Bcl-2, and anti-p-BclII were purchased from ProteinTech (Shanghai, China). Goat anti-rabbit and anti-mouse IgG–horseradish peroxidase (HRP) were purchased from Cell Signaling Technology (Beverly, MA).

### Strains and cell culture.

RAW 264.7 cells were stocked by the Key Laboratory of the Ministry of Education for Conservation and Utilization of Special Biological Resources in Western China. M. ovipneumoniae Queensland strain Y98 (M. ovipneumoniae Y98) was purchased from the China Institute of Veterinary Drug Control (Beijing, China). M. ovipneumoniae was cultured in a mycoplasma broth containing mycoplasma broth base CM403, supplementary reagent SR59 (Oxoid, Hampshire, UK), 0.5% glucose, and 0.002% phenol red at 37°C with 5% CO_2_ (48).

### Western blotting.

Total protein was extracted from RAW 264.7 cells using cell total protein extraction kit (KeyGen Biotech, Nanjing, China), and bicinchoninic acid (BCA) assay (protein assay kit; KeyGen Biotech, Nanjing, China) was used to quantify protein concentration. Protein samples (50 μg per lane) were separated via an SDS-PAGE on a 12% Tris-HCl polyacrylamide gel and transferred onto polyvinylidene difluoride (PVDF) membranes (EMD Millipore). Membranes were blocked with 5% skim milk solution, incubated with primary antibodies at room temperature for 1 h, and then incubated overnight at 4°C with the following primary antibodies: anti-β-actin (1:1,000), anti-LC3 (1:1,000), anti-NOD1 (1:1,000), anti-NOD2 (1:1,000), anti-Atg5 (1:1,000), anti-beclin1 (1:1,000), anti-JNK1/2 (1:1,000), anti-p-JNK1/2 (1:1,000), anti-Bcl-2 (1:1,000), and anti-p-BclII (1:1,000) diluted in block buffer. Following primary antibody incubation, the membranes were incubated with IgG-HRP secondary antibody (1:5,000) for 1 h at room temperature. Protein bands were visualized with an enhanced chemiluminescence (ECL) kit purchased from Bio-Rad (Hercules, CA) and detection reagent and analyzed using a GE Amersham Imager 600.

### Immunofluorescence microscopy.

We seeded 1 × 10^6^ RAW 264.7 cells on coverslips in 12-well plates. The cells were infected with M. ovipneumoniae (multiplicity of infection [MOI] of 10) for 12 h. The samples were washed twice with phosphate-buffered saline (PBS) and fixed with 4% paraformaldehyde at room temperature for 30 min, washed three times with PBS, and incubated with blocking buffer (3% bovine serum albumin) three times for 5 min at room temperature. The samples were permeabilized in 0.1% Triton X-100 (Sigma-Aldrich) and washed three times with PBS, followed by treatment with primary antibodies and LC3 (1:1,000) 1 h at room temperature; the samples were then washed three times for 5 min with blocking buffer and stained with fluorescein isothiocyanate (FITC)-labeled IgG for 1 h at room temperature. DNAs of macrophage were stained with DAPI (4,6-diamidino-2-phenylindole; Sigma-Aldrich). Fluorescent images were obtained using a confocal microscope (DM2500; Leica, Germany).

### RNA interference.

Small interfering RNA (siRNA) against the NOD2 and negative-control siRNA were purchased form Hanheng Biotechnology Co., Ltd. (Shanghai, China). The final concentration of siRNA was diluted in Opti-MEM medium. The cells were transfected with siRNA using lipofectamine 2000 in accordance with the manufacturer’s protocol. After 24 h, the mRNA level of NOD2 was measured by RT-PCR, and the protein expression of NOD2 was determined by Western blot analysis.

### Quantitative real-time PCR.

For quantitative real-time PCR (RT-PCR), total RNAs were purified from RAW 264.7 cells using TRIzol reagent. RNA was transcribed to cDNA with PrimerScript RT reagent kit. Then, RT-PCR was performed in a 20-μl reaction mixture containing SYBR green PCR master mix (TaKaRa), cDNA, and 0.2 mmol/liter of each primer at 95°C for 10 min and 40 cycles at 95°C for 10 s and 60°C for 45 s. The relative gene expression was normalized to internal control β-actin. Primer sequences for SYBR green probes of targets are follows: NOD2, forward, 5′-GGGCACCTGAAGTTGACATT-3′, and reverse, 5′-CCGACATATCCCACAGAGTT-3′, and β-actin, forward, 5′-TGAGAGGGAAATCGTGCGTGACAT-3′, and reverse, 5′-ACCGCTCGTTGCCAATAGTGATGA-3′.

### CCU assay.

The amount of M. ovipneumoniae in the cells is detected by color-changing units (CCU). Briefly, RAW 264.7 cells were collected by 2,000 × *g* centrifugation for 3 min and then washed with PBS three times. The cells were lysed by adding 1 ml autoclaved water for 10 min with gradual dilution to the first nine tubes. The tenth tube was used as a negative control without test solution (49). All the tubes were incubated at 37°C with 5% CO_2_ for 7 days to calculate the amount of M. ovipneumoniae in 1 ml.

### Statistical analyses.

In this study, all the data collected were obtained from at least three independent experiments for each condition. SPSS version 17.0 (IBM Corporation, USA) was used for descriptive statistics, and statistical evaluation of the data was performed by one-way analysis of variance (ANOVA) for comparisons of differences between two groups. *P* < 0.05 was considered to be statistically significant. Data were presented as the mean ± standard deviation (SD).
